# Supplementary material for: The Impact of Explanations on Layperson Trust in Artificial Intelligence–Driven Symptom Checker Apps: Experimental Study
Source: J Med Internet Res. 2021 Nov 3;23(11):e29386. doi: 10.2196/29386 (PMC8600426; doi:10.2196/29386)
Supplement: Multimedia Appendix 1 [file jmir_v23i11e29386_app1.docx]

## Appendix A

### Surveyed Symptom Checkers

Methodology: SCs were surveyed between April 2019 - July 2019. SCs were selected by searching the US and UK Android and iOS app stores for ``symptom checkers''. A Google search was also conducted from the UK for ``symptom checkers''. To be included an SC had to satisfy all of the following criteria:

- Be in the English language
- Be available in the UK or US
- Generate an output of a diagnosis, or triage instructions, or both
- Describe its mechanism as using a form of algorithm
- Identify as a SC (i.e. Google Search, whilst having the potential to generate diagnoses was not included as it is not presented as a tool for diagnosing oneself)

The selected SCs were investigated by entering symptoms closest aligned to the study's migraine diagnostic pathway. Results are classified based on subjective interpretation of the results' alignment to the identified explanatory ML mechanisms.

| Application | Function | Text Presented | Explanation Type |
| --- | --- | --- | --- |
| Ada Health | Diagnosis & Triage | People with symptoms similar to yours may require prompt medical assessment and care. You should seek advice from a doctor within the next few hours.  Possible causes  **1) Migraine**  Seek medical advice  3 out of 10 people with these symptoms had this condition.  **2) Tension-type headache**  Can usually be managed at home  Fewer than one out of 10 people with these symptoms had this condition.  **3) Shingles**  Seek medical advice  Fewer than one out of 10 people with these symptoms had this condition. | Social Proof |
| Apple | Covid-19 Checker | Based on your answers, you should talk to a doctor or a medical professional about your current symptoms. | No Explanation |
| Babylon Health | Diagnosis & Triage | People with symptoms similar to yours usually have the following condition:  **Migraine** 8 out of 10 people with these symptoms are likely to have this condition (a severe headache often accompanied by nausea or sensory changes)  This usually requires seeing a GP  Other possible causes of these symptoms include:  **Panic attack**  Less than 1 in 10 people with these symptoms are likely to have this condition (a sudden period of intense fear and anxiety).  This can usually be treated at home.  **Cluster headache**  Less than 1 in 10 people with these symptoms are likely to have this condition (a recurrent attack of severe headaches, usually one sided and often around the eye). | Social Proof |
| Babylon Health | Covid-19 Checker | The symptoms you mentioned sound worrying and could be caused by coronavirus (COVID-19).  Call 999 for an ambulance immediately and let them know you may have symptoms of coronavirus. | No Explanation |
| Buoy | Diagnosis & Triage | Normal occurrence of headache.  Headaches are extremely common and a primary reason people miss work or school. In many cases, the cause for headache is unknown. If your headache continues, changes or worsens, check your symptoms with us again or call your primary care doctor’s office. Your headache looks like a normal variation and can be treated at home with an over-the-counter pain reliever.  Reasons for:  This could explain your headache  Reasons against:  This wouldn’t explain your irritability | Input Influence |
| Health Tap | Diagnosis & Triage | 35 out of 100 people with your symptoms have: **Tension headache**  Usually requires talking to a doctor  35 of 100 people with your symptoms have: **Cluster headache**  Usually requires talking to a doctor urgently  8 of 100 people with your symptoms have: **Migraine**  Usually requires talking to a doctor | Social Proof |
| Isabel | Diagnosis | Possible causes  Symptoms: Headache for a few hours, light sensitive, sick  Superior Canal Dehiscence Syndrome  Migraine (common)  Cyclic Vomiting Syndrome  Concussion (red flag, common)  Tickborne Encephalitis  Visual Stress Delirium Tremens (red flag)  Anterior Uveitis | Social Proof |
| K-Health | Diagnosis & Triage | Based on what you told me here’s how 456 cases like yours turned out.  Tension type headache 63%  The most common type of headache | Social Proof |
| Symptomate | Diagnosis & Triage | Food poisoning  Weak evidence  Explanation  Reasons for:   - Headache Severe headache - Headaches history shorter than 3 months   Reasons against:   - Fever - Dizzy | Input Influence |
| YourMD | Diagnosis & Triage | Possible causes:  Tension headache  This condition can be managed with self-care  Reported as present:   - Headache - Severe headache - Eyes sensitive to light - Headache at the front of head   Report as absent:   - Pain on both sides of the head - Tender scalp - Bulging or protruding eye - Headache on waking up - Vomiting | Input Influence |
