# Supplementary material for: The Impact of Explanations on Layperson Trust in Artificial Intelligence–Driven Symptom Checker Apps: Experimental Study
Source: J Med Internet Res. 2021 Nov 3;23(11):e29386. doi: 10.2196/29386 (PMC8600426; doi:10.2196/29386)
Supplement: Multimedia Appendix 2 [file jmir_v23i11e29386_app2.docx]

Table S2: Survey questions used to measure attitudes of trust in explanation

| Variable | Question | Category | Intended Measurement |
| --- | --- | --- | --- |
| Q16_L | The symptom checker should be trusted. | Faith | Blind trust |
| Q17_L | The symptom checker is safe. | Faith |  |
| Q18_L | The symptom checker is reliable. | Faith |  |
| Q19_L | I am wary of the symptom checker. | Faith |  |
| Q20_L | The user should follow the advice the app gave them. | Reliance | Reliance (i.e. would you perform an action based on the result and explanation provided?) |
| Q21_L | If I felt unwell in the future, I’d use this symptom checker. | Reliance |  |
| Q22_L | The symptom checker is a helpful tool for people when they feel unwell. | Reliance |  |
| Q23_L | The explanation of why the symptom checker suggests the illness is satisfying. | Satisfaction | Satisfaction |
| Q24_L | The explanation of why the symptom checker suggested the illness it did went into enough detail. | Satisfaction |  |
| Q25_L | It is clear to me why the symptom checker suggested this particular illness. | Satisfaction |  |
| Q26_L | I understand how the symptom checker arrives at a diagnosis. | Comprehension | Investigating comprehension and also user’s mental model of explanation |
| Q27_L | It’s easy to follow what the system does. | Comprehension |  |
| Q28_L | I understand why the symptom checker suggested the illness it did. | Comprehension |  |

Table S3: Survey Questions Used to Gather Contextual Information

| Variable | Question | Category | Intended Measurement | Response |
| --- | --- | --- | --- | --- |
| Disease_Seriousness | How serious is the disease the symptom checker diagnosed? | Seriousness | Severity perception | Very serious, Moderately serious, Not very serious |
| Headache_Experience | Have you ever had a headache? | Past Medical History | Experience of head pain | No, Yes, Yes (within the past 12 months) |
| When_Use | When would you use a symptom checker like this? | Tech adoption propensity | Use propensity | Any time I felt poorly, If I felt moderately unwell, If I couldn’t speak to a human clinician, In situations where I would currently Google my symptoms, I would never use this kind of symptom checker, Don’t know |
| Used_SC_Recently | Have you personally used a symptom checking application in the last 12 months? | Tech adoption propensity | Familiarity with SCs | Yes, No, Don’t know |
| Like_New_Tech | I like using new technologies. | Tech adoption propensity | Technology adoption propensity | Likert |
| Smartphone_Reliance | I would struggle to spend a day without my smartphone. | Tech adoption propensity | Technology adoption propensity | Likert |
