# Supplementary material for: The Impact of Explanations on Layperson Trust in Artificial Intelligence–Driven Symptom Checker Apps: Experimental Study
Source: J Med Internet Res. 2021 Nov 3;23(11):e29386. doi: 10.2196/29386 (PMC8600426; doi:10.2196/29386)
Supplement: Multimedia Appendix 3 [file jmir_v23i11e29386_app3.docx]

Table S4: Multivariate and univariate analyses of variance of trust in explanation by varying disease presented.

| Explanation | Parametric MANOVA | | |  | Parametric *t*-test |  | | | | | | | | |
| --- | --- | --- | --- | --- | --- | --- | --- | --- | --- | --- | --- | --- | --- | --- |
| Type |  | | |  | Faith | Comprehension | Depth | | | | | | |  |
|  | *F V* | *df* | *p* | | *t df p* | *d β t df p d* | *β* |  | *t* | *df* | *p* | *d* | *β* |  |
| Input | 6.81 .068 | 2,186 | .001 | | 2 159 .046 | .29 .509 -1 185 .250 .17 | .515 |  | / | / | / | / | / |  |
| Social Proof | 2.68 .043 | 3,179 | .049 | | 2 181 .102 | .24 .507 -1 181 .330 .14 | .529 |  | .9 | 176 | .357 | .14 | .536 |  |
| Counterfactual | 2.98 .031 | 2,189 | .053 | | -.3 184 .743 | .05 .756 -2 186 .031 .31 | .506 |  | / | / | / | / | / |  |
| No Explanation | 4.26 .066 | 3,184 | .006 | | -.5 182 .605 | .03 .653 -3 182 .002 .46 | .530 |  | -.2 | 183 | .850 | .03 | .853 |  |

Note: Total *N* = 750, Input Influence *N* = 189, Social Proof *N* = 183, Counterfactual *N* = 192, No Explanation *N* = 186. *F* is the MANOVA test statistic, *V* is Pillai’s Trace, *df* is the number of degrees of freedom, *p* is the significance level (i.e. *p* value), *t* is the test statistic of the *t* test, and *β* is the power. “/” indicates where a factor was not generated by analysis.
